# Elevated Levels of Toxic Bile Acids in Serum of Cystic Fibrosis Patients with *CFTR* Mutations Causing Pancreatic Insufficiency

**DOI:** 10.3390/ijms232012436

**Published:** 2022-10-18

**Authors:** Harold Tabori, Jochen Schneider, Stefan Lüth, Carlos Zagoya, Anton Barucha, Thomas Lehmann, Eberhard Kauf, Astrid Barth, Jochen G. Mainz

**Affiliations:** 1 Cystic Fibrosis Center, Brandenburg Medical School (MHB) University, Klinikum Westbrandenburg, 14770 Brandenburg an der Havel, Germany; 2 Internal Medicine, Alexianer Hedwigshöhe Hospital, 12526 Berlin, Germany; 3 Cystic Fibrosis Centre, Jena University Hospital, 07740 Jena, Germany; 4 Department of Gastroenterology, Faculty of Health Sciences Brandenburg, Brandenburg Medical School Theodor Fontane, 14770 Brandenburg an der Havel, Germany; 5 Faculty of Health Sciences, Joint Faculty of the Brandenburg University of Technology Cottbus-Senftenberg, Brandenburg Medical School Theodor Fontane, University of Potsdam, 14469 Potsdam, Germany; 6 Institute of Medical Statistics, Jena University Hospital, 07743 Jena, Germany; 7 Institut für Pharmakologie und Toxikologie, Jena University Hospital, 07743 Jena, Germany

**Keywords:** cystic fibrosis, CF liver disease, hepatic, biliary, bile acid, high performance liquid chromatography

## Abstract

Hepatobiliary involvement is a hallmark in cystic fibrosis (CF), as the causative CF Transmembrane Conductance Regulator (CFTR) defect is expressed in the biliary tree. However, bile acid (BA) compositions in regard to pancreatic insufficiency, which is present at an early stage in about 85% of CF patients, have not been satisfactorily understood. We assess the pattern of serum BAs in people with CF (pwCF) without CFTR modulator therapy in regard to pancreatic insufficiency and the *CFTR* genotype. In 47 pwCF, 10 free and 12 taurine- and glycine-conjugated BAs in serum were prospectively assessed. Findings were related to genotype, pancreatic insufficiency prevalence (PIP)-score, and hepatic involvement indicated by serum liver enzymes, as well as clinical and ultrasound criteria for CF-related liver disease. Serum concentrations of total primary BAs and free cholic acid (CA) were significantly higher in pwCF with higher PIP-scores (*p* = 0.025, *p* = 0.009, respectively). Higher total BAs were seen in pwCF with PIP-scores ≥0.88 (*p* = 0.033) and with pancreatic insufficiency (*p* = 0.034). Free CA was higher in patients with CF-related liver involvement without cirrhosis, compared to pwCF without liver disease (2.3-fold, *p* = 0.036). pwCF with severe *CFTR* genotypes, as assessed by the PIP-score, reveals more toxic BA compositions in serum. Subsequent studies assessing changes in BA homeostasis during new highly effective CFTR-modulating therapies are of high interest.

## 1. Introduction

**Figure 1:**
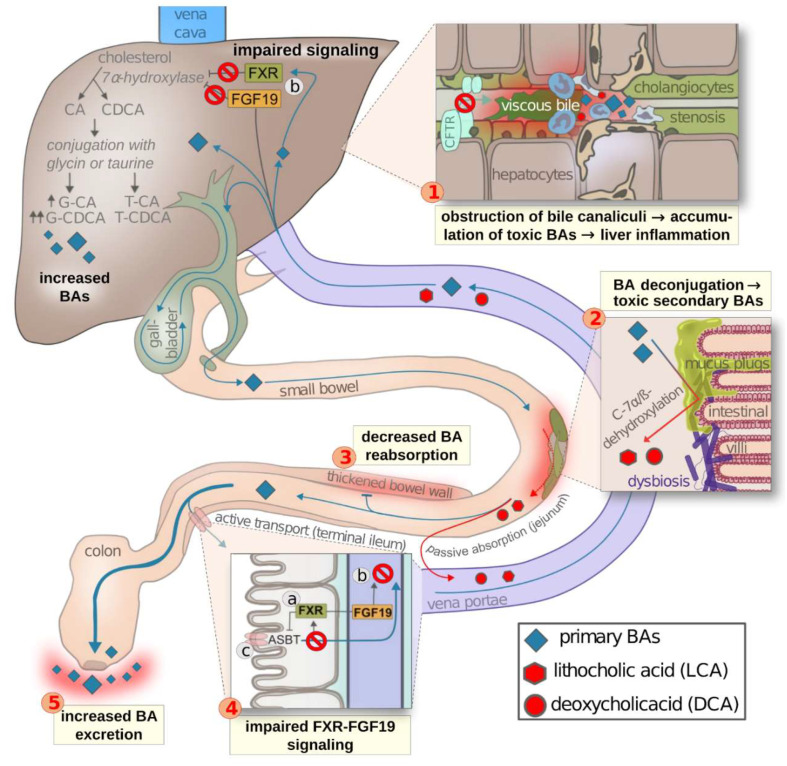
Physiological bile acid production and enterohepatic circulation, including proposed mechanisms of alterations related to CFTR deficiency (in red). (**1**) CFTR dysfunction on the apical side of the cholangiocytes leads to secretion of bile with high viscosity, occluding the bile canaliculi. Cystic fibrosis liver disease (CFLD) is caused by accumulation of hydrophobic, toxic, glycine-conjugated BA promoting neutrophil activation and inflammation, which damages hepatocytes and bile ducts. (**2**) Mucus plugs and dysbiosis due to increased acidity, antibiotic use, and swallowed contaminated saliva promote deconjugation of BAs, resulting in higher concentrations of toxic secondary BAs (LCA, DCA) and decreased enteric BA reabsorption (enterohepathic circulation). (**3**) Impaired BA resorption caused by bowel wall thickening. (**4**) (**a**) Active resorption of BAs activates FXR, stimulating the synthesis of FGF19. (**b**) FGF19 exerts negative feedback on 7-alpha-hydroxylase, the key enzyme in BA synthesis. (**c**) FXR activation is also hypothesized to down-regulate ASBT channels. BA malabsorption in CF results in impaired FXR-FGF19 signaling. (**5**) Increased fecal BA excretion in CF (BA losses). BA: bile acid; ASBT: apical sodium-dependent bile acid transporter; FXR: farnesoid X receptor; FGF19: fibroblast growth factor 19; LCA: lithocholic acid; DCA: deoxycholic acid; CA: cholic acid; G-CA: glycocholic acid; T-CA: taurocholic acid; CDCA: chenodeoxycholic acid; G-CDCA: glycochenodeoxycholic acid; T-CDCA: taurochenodeoxycholic acid. [Image by Anton Barucha].

Cystic fibrosis (CF) is the most frequent life-threatening inherited disease in populations with Caucasian descent, characterized by multi-organ involvement due to impaired ion transport in apical membranes of the exocrine glands [1]. Over the last decades, pulmonary disease has been in scientific and clinical focus, as about 90% of people with CF (pwCF) die prematurely from pulmonary destruction. With a marked improvement in survival, the involvement of other organs, including hepatobiliary pathology, is currently coming into focus, as the underlying CF Transmembrane Conductance Regulator (CFTR) defect is equally expressed in biliary ducts and gallbladder epithelia. As a result, hepatobiliary involvement is the third most frequent cause of premature death in pwCF [2]. Whereas CF-related liver disease (CFLD) generally manifests asymptomatically, the most common histopathological findings are: focal biliary cirrhosis, liver steatosis, secondary sclerosing cholangitis, and liver cirrhosis leading to portal hypertension as the most critical manifestation [3]. Furthermore, functional disorders of the gallbladder, such as micro-gallbladder, are frequent. An additional manifestation of hepatobiliary involvement in CF is an abnormal bile acid (BA) metabolism, including an impaired hepatobiliary circuit, resulting in malabsorption of nutrients and excessive fecal bile acid excretion (see Figure 1). However, the pathophysiology of this crucial biochemical process is, to date, not sufficiently understood [4,5].

BAs are primarily regarded as detergents, as their central function is to eliminate cholesterol from the body via the intestinal lumen and feces. BAs also play a key role in the solubilization, digestion, and absorption of dietary lipids, as well as lipid-soluble vitamins. As recently demonstrated, BAs also act as signaling molecules in liver regeneration after partial hepatectomy and partial liver transplantation [6,7,8]. Primary BAs (chenodeoxycholic acid (CDCA) and cholic acid (CA)) are de novo synthesized from cholesterol by hepatocytes in the liver as a result of hydroxylation processes at carbon positions of different steroid nuclei. After their synthesis in the liver and before being secreted into the intestine, free primary BAs conjugate with glycine or taurine, thereby increasing their water solubility (hydrophilicity—low pKa values) and, consequently, resulting in bile acid anions. Such an increase in BAs’ solubility facilitates their return to the liver, either by passive absorption across the entire small intestine or active transport in the terminal ileum [9,10,11].

In the small bowel, conjugated BAs become metabolized by bile salt hydrolase enzymes to release unconjugated and more hydrophobic BAs, which may be excreted with the feces or biotransformed into more toxic secondary BA species [11]. Differences in intestinal bacterial flora composition induce variations in bile salt composition [9]. In healthy individuals, approximately 95% of BAs are reabsorbed during their passage through the intestine and returned to the liver as part of the enterohepatic circulation [12,13,14]. Reabsorption occurs through active transport in the terminal ileum by the apical sodium-dependent bile salt transporter (ASBT) and by passive diffusion along the entire axis of the intestine [15]. After reabsorption, the remaining 5% of BAs becomes substrate for significant microbial biotransforming reactions in the large bowel [16] or is excreted in feces [13].

Many factors are directly involved in BA malabsorption in CF such as (see Figure 1): defective CFTR channels [17], small intestine bacterial overgrowth (SIBO) [9], increased BA losses, decreased BA resorption in the terminal ileum, and an impaired BA interaction with the hepatic and intestinal farnesoid X receptor (FXR), which modulates cholesterol 7α-hydroxylase (CYP7A1), the rate-limiting enzyme in BA synthesis [5,18,19]. To date, however, the exact underlying mechanism of BA malabsorption remains unknown.

In general, pwCF reveal a more toxic BA profile, which may be caused by the inherently altered viscous mucoid secretion in bile ducts and the consequent retention of cytotoxic BAs. Although still debatable, pwCF have been reported to show higher levels of primary and secondary BAs [20], which are potentially more toxic due to increased deconjugation by the altered intestinal flora [21], as compared to healthy controls. On the other hand, the observation of abnormally high fecal excretion of BAs together with the similarity in duodenal BA concentrations found in pwCF and controls may imply an increase in de novo BA synthesis in the liver of pwCF [22].

High levels of hydrophobic BAs have been hypothesized to contribute to the development of CFLD [5]. In addition, the identification of non-CFTR genetic polymorphism SERPINA1 Z allele was mentioned as a risk factor of liver disease in CF [23]. More recently, only one study has explored the association between BA concentrations in serum and the degree of liver involvement (LI) in pwCF, wherein it is suggested that serum deoxycholic acid and its glycine conjugate have the potential to serve as biomarkers to differentiate between pwCF with non-cirrhotic LI and pwCF with no detectable liver disease [24]. Nevertheless, there is a lack of studies investigating the relationship between *CFTR* genotype/phenotype and BAs observed in pwCF.

The objective of this study was to assess the composition patterns of free, taurine- and glycine-conjugated BAs from pwCF in regard to exocrine pancreatic insufficiency, according to recently defined *CFTR* genotype (pancreatic insufficiency prevalence [PIP] score) and CFLD classifications [4,25,26]. This allows for assessment of the role of CF patients’ *CFTR* genotype and phenotype in BA homeostasis.

## 2. Results

### 2.1. Demographic Characteristics

Forty-seven pwCF (46.8% females) were prospectively enrolled. The mean age in the participants was 18.9 ± 12.8 years. PI was present in 42 pwCF (89.4%) at inclusion, 14 pwCF (30.4%) revealed CFLD according to criteria defined in 2011 by Debray et al. [26], and 4 pwCF (8.7%) had been diagnosed with liver cirrhosis. *CFTR* mutations in both alleles were identified in 43 out of the 47 pwCF included (91.5%). Further characteristics of the participants are presented in Table 1. Adequate visualization of the pancreas by ultrasound could be achieved in 95% of the pwCF (45/47), 95% of whom revealed pancreatic lipomatosis (43/45).

**Figure 2:**
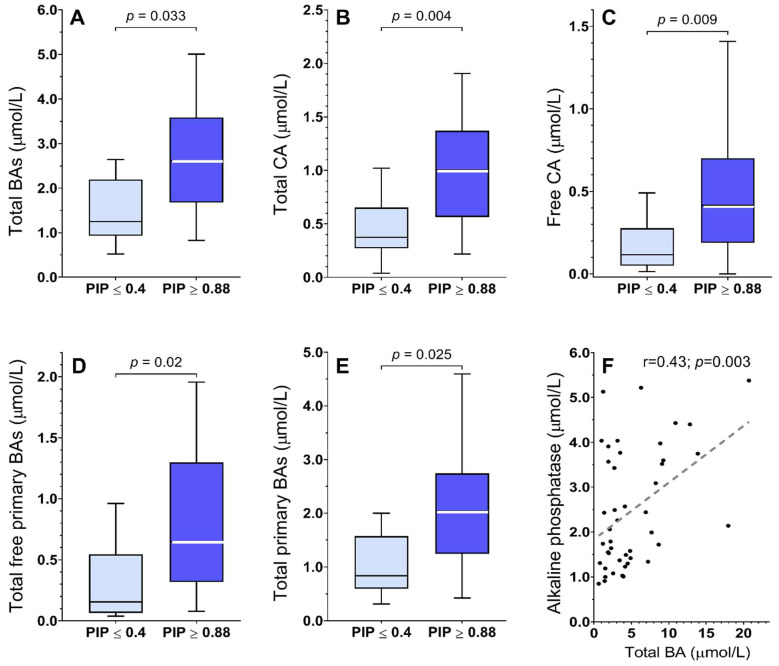
Primary bile acid (BA) concentrations in pwCF classified according to their pancreatic insufficiency prevalence (PIP) score. (**A**): Concentration of total BAs. (**B**): Total cholic acid (CA) concentrations. (**C**): Free cholic acid (CA) concentrations. (**D**): Total concentrations of free primary BAs, i.e., CA+CDCA. (**E**): Total concentrations of all primary BAs, i.e., CA+G-CA+T-CA+ CDCA+G-CDCA+T-CDCA. (**F**): Significant correlation between the total concentration of bile acids and alkaline phosphatase.

Median concentration of serum biochemical parameters alanine aminotransferase (ALT), aspartate aminotransferase (AST), γ-glutamyl transpeptidase (γ-GT), alkaline phosphatase (AP), and glutamate dehydrogenase (GLDH) resulted in (median, [Q_1_, Q_3_]) 0.41 (0.27, 0.69) µmol/L, 0.36 (0.30, 0.51) µmol/L, 0.23 (0.14, 0.46) µmol/L, 2.06 (1.34, 3.68) µmol/L, and 50.00 (28, 71) µmol/L, respectively. The median concentration of total BAs was (median, [Q_1_, Q_3_]): 2.1 (1.3, 3.6) µmol/L. A correlation between AP and the total BA concentration was found to be significant (r = 0.43; *p* = 0.003) (Figure 2F). However, no correlation was observed between BA concentrations and the 17 CF-relevant pathologies examined by abdominal US. Although tertiary BAs were included in the quantification of total BA concentrations, the concentration of each tertiary BA showed no association with *CFTR* genotype or phenotype classifications.

### 2.2. Bile Acids in pwCF in Relation to CFTR Genotype and Phenotype

**Figure 3:**
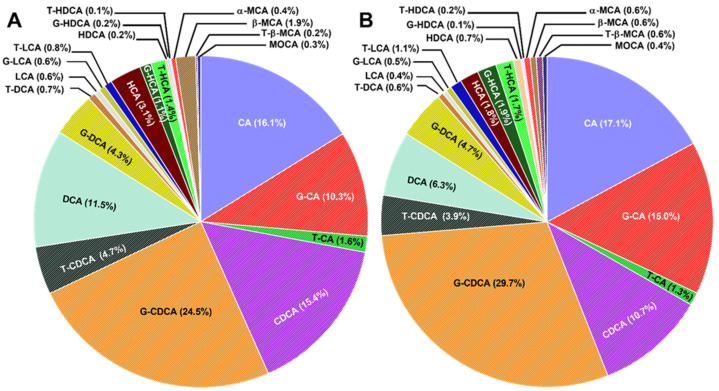
Bile acid distribution in pwCF with (**A**) a PIP score ≤ 0.4 (mild *CFTR* genotype) and (**B**) a PIP score ≥ 0.88 (severe *CFTR* genotypes). CA: cholic acid; G-CA: glycocholic acid; T-CA: taurocholic acid; CDCA: chenodeoxycholic acid; G-CDCA: glycochenodeoxycholic acid; T-CDCA: taurochenodeoxycholic acid; DCA: deoxycholic acid; G-DCA: glycodeoxycholic acid; T-DCA: taurodeoxycholic acid; LCA: lithocholic acid; G-LCA: glycolithocholic acid; T-LCA: taurolithocholic acid; HDCA: hyodeoxycholic acid; G-HDCA: glycohyodeoxycholic acid; T-HDCA: taurohyodeoxycholic acid; HCA: hyocholic acid; G-HCA: glycohyocholic acid; T-HCA: taurohyocholic acid; α-MCA: α-murocholic acid; β-MCA: β-muricholic acid; MOCA: murocholic acid; T-β-MCA: tauro-β-muricholic acid.

**Figure 4:**
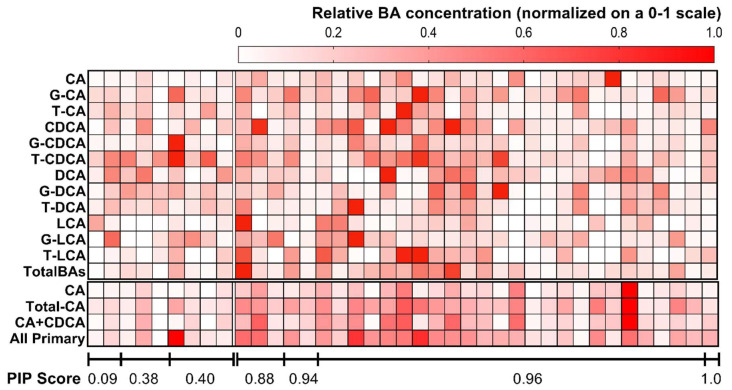
Distribution of bile acids according to each patient’s (*x*-axis) pancreatic insufficiency prevalence (PIP) score (mild: ≤0.40 vs. severe: PIP > 0.40). In this figure, for illustrative purposes, concentrations of bile acids (color scale) are normalized on a zero (minimum) to one (maximum) scale. CA: cholic acid; G-CA: glycocholic acid; T-CA: taurocholic acid; CDCA: chenodeoxycholic acid; G-CDCA: glycochenodeoxycholic acid; T-CDCA: taurochenodeoxycholic acid.

Bile acid distributions in pwCF with mild and severe *CFTR* genotypes are shown in Figure 3 and Figure 4. Therein, it can be seen that G-CDCA is predominant in both groups, followed by CA. A slightly higher amount of G-CDCA was observed in pwCF with the severe *CFTR* genotype (29.6% vs. 24.5%).

Concentrations of total BAs in serum were significantly higher in pwCF with severe *CFTR* genotypes (2.1-fold; *p* = 0.033), as measured by the PIP score. More specifically, free CA concentrations were found to be 3.4-fold higher in pwCF with severe *CFTR* genotypes (*p* = 0.009). In a similar way, total CDCA tended to be lower in pwCF with mild *CFTR* genotypes, although this result did not attain statistical significance (0.5-fold; *p* = 0.123). Correspondingly, G-CA tended towards higher values in pwCF with severe *CFTR* genotypes without reaching statistical significance (1.5-fold; *p* = 0.123). The sum of all CA concentrations, i.e., CA+G-CA+T-CA, was 2.7-fold higher in pwCF with severe *CFTR* genotypes than in those with the mild *CFTR* genotype (*p* = 0.004) (Figure 2 and Table 2).

Additionally, the concentration of total free primary BAs, i.e., CA+CDCA, in serum was significantly higher in pwCF with severe *CFTR* genotypes (4.3-fold; *p* = 0.020). In general, total primary BAs, i.e., CA+G-CA+T-CA+CDCA+G-CDCA+T-CDCA, were found to be higher in pwCF with severe *CFTR* genotypes (2.4-fold; *p* = 0.025). The two ratios of free CA/CDCA and conjugated CA/CDCA had a tendency towards higher values in pwCF with severe *CFTR* genotypes (1.9-fold; *p* = 0.299 and 1.3; *p* = 0.566, respectively). Similarly, the sum of glycine-conjugated CA and CDCA (G-CA+G-CDCA) tended towards higher values in pwCF with severe *CFTR* genotypes (2.0-fold; *p* = 0.069) (Figure 2 and Table 2). In contrast, the ratio between taurine-conjugated CA and CDCA (T-CA/T-CDCA) had a tendency towards higher values in pwCF with mild *CFTR* genotypes compared to pwCF with severe *CFTR* genotypes (1.3-fold; *p* = 0.755). 

Notably, the G:T ratio (defined as the ratio between G-CA+G-CDCA+G-LCA+G-DCA and T-CA+T-CDCA+T-LCA+T-DCA) was significantly higher in the severe *CFTR* genotype subgroup (3.1-fold; *p* < 0.01). 

Secondary BA differences in relation to *CFTR* genotype did not reach significance (Table 3). Regarding liver function tests, AST and AP levels were higher in the CF subgroup with severe *CFTR* genotypes (*p* = 0.022 for both). Although ALT, GLDH, and γ-GT tended to show higher values in the severe *CFTR* genotype cohort, those changes did not attain statistical significance (Table 4).

**Figure 5:**
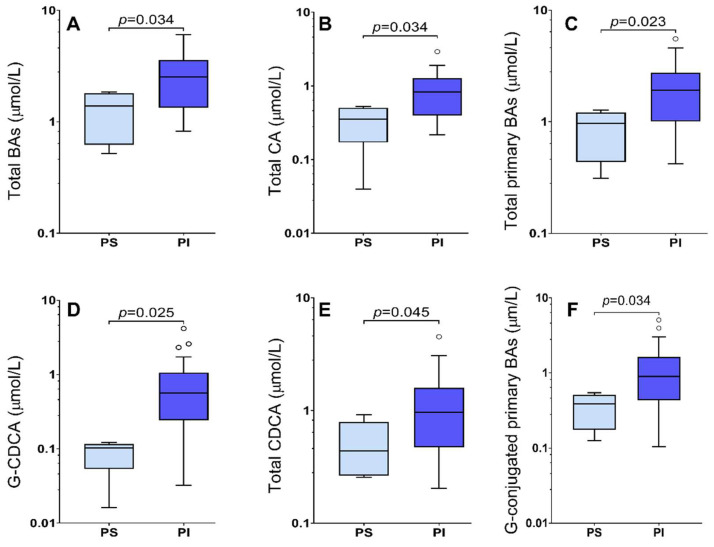
Bile acid concentrations from pwCF classified according to their pancreatic sufficiency status: pancreatic sufficiency (PS) or pancreatic insufficiency (PI). (**A**) Total bile acids. (**B**) Total CA, i.e., CA+T-CA+G-CA. (**C**) Total primary BAs, i.e., CA+T-CA+G-CA+ CDCA+T-CDCA+G-CDCA. (**D**) Glycochenodeoxycholic acid (G-CDCA). (**E**) Total chenodeoxycholic acid, i.e., CDCA+T-CDCA+G-CDCA. (**F**) Glycine-conjugated primary BAs, i.e., G-CA+G-CDCA. Circles represent data points above the value Q_3_ + 1.5 × (Q_3_-Q1).

Levels of total BAs in pancreatic-sufficient pwCF were lower than in pancreatic-insufficient pwCF (median, [Q_1_, Q_2_]: 1.38 (0.62, 1.80) µmol/L vs. 2.53 (1.34, 3.59) µmol/L; *p* = 0.034) (Table 5 and Figure 5). Similarly to the group with severe *CFTR* genotype, total CA, total primary BAs, total BAs, and the G:T ratio were significantly elevated in pwCF with PI status (Table 5 and Figure 5). In addition to that, total CDCA, i.e., CDCA+G-CDCA+T-CDCA, was significantly higher in the subgroup with PI status than in the pancreatic-sufficient subgroup. Furthermore, significantly higher levels of AST were observed in pwCF with PI (0.39 (0.31, 0.56) vs. 0.21 (0.19, 0.37); *p* = 0.033), whereas the other parameters tended to be elevated without reaching statistical significance.

### 2.3. Bile Acids in Relation to CF Liver Disease (CFLD)

**Figure 6:**
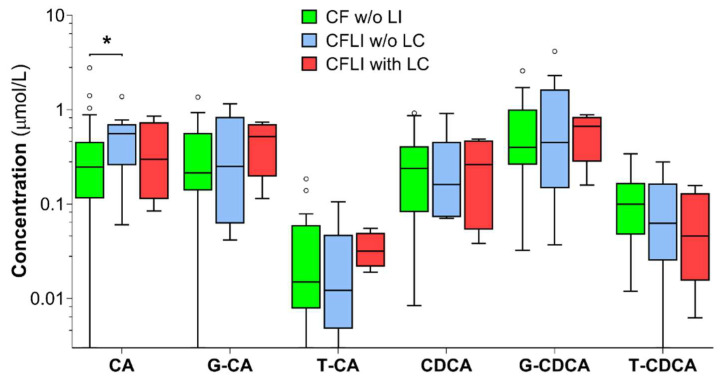
Serum primary bile acids distribution in the three subgroups classified according to pwCF’s CF-related liver involvement: cystic fibrosis without evidence of liver involvement (CF *w*/*o* LI) (*n* = 32), cystic fibrosis-related liver involvement without cirrhosis (CFLI *w*/*o* LC) (*n* = 10), and cystic fibrosis-related liver involvement with cirrhosis (CFLI with LC) (*n* = 4). Circles represent data points above the value Q_3_ + 1.5 × (Q_3_-Q1). * *p* = 0.036; CA: cholic acid; G-CA: glycocholic acid; T-CA: taurocholic acid; CDCA: chenodeoxycholic acid; G-CDCA: glycochenodeoxycholic acid; T-CDCA: taurochenodeoxycholic acid.

Free CA was significantly higher in the CFLI *w*/*o* LC subgroup, compared to the CF *w*/*o* LI subgroup (2.3-fold; *p* = 0.036). G-CDCA was elevated in the CFLI *w*/*o* LC subgroup and was higher than that in pwCF *w*/*o* LI, but lower than in the CFLD with LC subgroup, although no significance was achieved for these comparisons. In all subgroups, median concentrations of free and glycine-conjugated primary BAs were higher than medians of taurine conjugates (Figure 6). Additionally, total BAs showed the highest values in the CFLI *w*/*o* LC subgroup. Furthermore, T-CA/T-CDCA was significantly elevated in the CFLD with LC subgroup compared to CF *w*/*o* LI (2.4-fold; *p* = 0.038) and CFLI *w*/*o* LC (2.8-fold; *p* = 0.036) subgroups. CA/CDCA and G-CA+G-CDCA showed the highest values in the CFLI *w*/*o* LC subgroup (Table 6).

Comparisons with respect to CFLD revealed that all liver function test parameters were significantly higher in pwCF with CFLD (*p* = 0.005, 0.033, 0.0002, 0.039, and 0.036 for ALT, AST, γ-GT, AP, and GLDH, respectively) (Table 4).

## 3. Discussion

In this prospective study, we assessed the association between serum BA levels in pwCF and the status of pancreatic insufficiency, represented clinically and by the pancreatic insufficiency prevalence (PIP) score. This surrogate measure classifies the severity of specific *CFTR* mutations, associating higher scores with pancreatic insufficiency (PI) and, conversely, lower scores to increased risks for pancreatitis. 

The complex pattern of bile acids in serum from pwCF associates increased total BA concentrations in pwCF with clinical pancreatic insufficiency and with higher PIP scores. Specifically, we found that higher PIP scores ≥ 0.88 are significantly associated with increased serum concentrations of total primary BAs. Particularly, free CA concentration was 3.4-fold higher when compared to concentrations in pwCF carrying a mild *CFTR* genotype. To our knowledge, this is the first study showing an association between *CFTR* genotype and BAs.

Our findings are supported by reports by Smith et al. [27] showing that histological markers of CF-related liver injury (severity of fibrosis and degree of inflammation) are significantly associated with elevation of CA. Similarly, Azer and colleagues found high levels of CA to be associated with progression of hepatic injury [28]. More recently, Drzymała et al. [24] observed higher CA concentrations in serum from pwCF compared to healthy subjects. In addition, the authors found CA concentrations to be higher in patients with some degree of liver involvement, including cirrhosis, than in pwCF without a diagnosis of liver disease.

Although we did not find a strong correlation between PIP scores, the pancreatic status, and CFLD, increased CA levels in the serum of pwCF with severe *CFTR* genotypes could be seen as a pro-inflammatory response and, consequently, as a risk for progression to CFLD. Furthermore, liver injury in CF with higher tissue permeability, more frequent in pwCF with more severe *CFTR* genotypes, may contribute to higher levels of bile acids in the serum of these pwCF [29].

Previously, elevated sums of G-CA+G-CDCA had been reported to be a marker for early hepatic allograft dysfunction in transplanted pwCF [30]. Interestingly, in our CF cohort, this sum tended to be elevated, accounting for almost 45% of BAs in pwCF with severe *CFTR* genotypes. Moreover, a significantly increased G:T ratio was observed in the severe *CFTR* genotype subgroup. As pointed out in previous studies, the predominance of toxic hydrophobic glycine conjugates [13,31] and, correspondingly, decreased taurine conjugates [32,33] could contribute to the maintenance of a potentially harmful cytotoxicity [13,21] and induce hepatocyte apoptosis [34].

The imbalance in the G:T ratio observed in pwCF with severe *CFTR* genotypes may, at least partially, derive from bowel wall abnormalities, an important factor impairing the enterohepatic circuit in pwCF [35]. In a previous study including abdominal ultrasound, higher rates of pathologies, including thickened bowel walls (TBW) > 4mm, were found in pwCF with PI and with more severe class I-III *CFTR* mutation [36]. Furthermore, taurine deficiency in pwCF with severe *CFTR* genotypes has been attributed to decreased BA resorption in the terminal ileum [37], a pathology supposedly more frequent in pwCF with TBW.

Similar to the results obtained with the PIP score, phenotype classification with regard to the pancreatic status revealed a more toxic BA pattern in pwCF with PI. This is to a large extent expected, as this classification is associated with *CFTR* genotype severity as measured with the PIP score.

Furthermore, the above-described toxic BA pattern associated with higher CA concentrations was also observed in pwCF with CF-related liver involvement (CFLI) *w*/*o* LC. This is in agreement with the results of Smith et al. [27] and O’Brien et al. [38], who proposed that pwCF with CFLI still preserve some residual liver function and, therefore, accumulate more BAs in the canaliculi obstructed with viscous bile [39]. Moreover, cirrhotic pwCF revealed, as expected, lower CA levels than the CFLI *w*/*o* LC group. A similar pattern was observed by Drzymała et al. [24], reporting lower CA levels in patients with liver cirrhosis compared to those without liver involvement. Compared to the CFLI *w*/*o* LC group, this appears to be a consequence of the impaired hepatic bile synthesis in cirrhosis. Accordingly, Vlahcevic et al. [40] found a reduction in CA and CDCA synthesis in patients with alcohol-related cirrhosis, concluding that a reduction in bile acid synthesis present in patients with cirrhosis is caused by both defective feedback control regulating bile acid synthesis and defective BA synthesis in the liver [40].

In cirrhotic pwCF, the T-CA/T-CDCA ratio was significantly higher than in the other subgroups, i.e., CF without LI and CFLI without LC. Analogously to G-CA+G-CDCA, this ratio was observed to be a marker of early hepatic allograft dysfunction [30]. To our knowledge, however, no further studies have been conducted validating those findings. The increased T-CA/T-CDCA ratio in cirrhotic pwCF may be a consequence of a decreased amount of T-CDCA in the BA pool, resulting from CDCA’s higher hydrophobicity [41] and, thus, higher toxicity. Other studies have postulated that bacteria of several genera have evolutionarily developed mechanisms to protect themselves from bile acid toxicity via bile salt hydrolase (BSH) activity [42,43,44]. According to this, BSH activity results in the transformation of BA into deconjugated BA species that are less toxic, resulting in less glycine conjugates and the apparent resistance of T-CDCA to being deconjugated by intestinal bacteria due to its lower toxicity (toxicity of glycine > taurine conjugates). Following this hypothesis, this would imply a decreased proportion of T-CDCA returning to the liver and, consequently, to the taurine pool in the intestine. However, given the limited sample size of cirrhotic pwCF considered herein, studies with larger subgroups of patients are necessary to assess this hypothesis. Further studies addressing the role of taurine supplements as a therapeutic approach to shift the BA pool to a less toxic pattern are lacking.

Other factors could be attributed to the complex etiology of the impaired BA metabolism in the enterohepatic circuit in CF, such as: (A) an impaired microflora (due to increased acidity, antibiotic use, and swallowed contaminated saliva) promoting BA deconjugation, more toxic secondary BAs (LCA, DCA) [20], and increased BA elimination; (B) a thickened bowel wall decreasing BA resorption in the terminal ileum; (C) increased BA excretion (BA losses); and (D) impaired FXR-FGF19 signaling by a defective feedback control regulating BA synthesis and, consequently, promoting BA accumulation. However, the exact mechanism remains unknown (see Figure 1). 

Altogether, the impaired BA pattern in pwCF with severe *CFTR* genotypes, characterized by increased CA and the predominance of glycine conjugates, appears to be related to more hepatotoxic effects contributing to the complex multifactorial etiology of CFLD [45].

Although a phenotype/genotype CFLD correlation has not yet been established, it was recently proposed that modifier genes contribute to the risk of severe CFLD. Risk factors such as class I-III mutations on both alleles, meconium ileus, and male gender have been identified as contributing to the development of liver involvement [46]. In line with this, according to Drzymała et al. [24], cirrhotic and non-cirrhotic liver involvement is characterized by several determinants, such as high BA levels and severe *CFTR* genotypes. Nevertheless, data regarding genotype severity have not yet been available for bile acid profiles. This field requires a better understanding in order to identify potential targets for modulating liver disease severity in CF [45]. 

## 4. Limitations

In terms of limitations, our results are being published many years after recruitment finalization and the analysis of the prospectively obtained serum samples. However, this delay allowed us to implement new categorizations of pwCF regarding CFLD criteria, as defined by Debray et al. in 2011 [26], and PIP scores, as defined by Ooi et al. in 2011 [25]. The delayed publication of these important classifications by Ooi et al. [25] and Debray et al. [26] demonstrates the lack of attention abdominal involvement received in previous decades, when pwCF tended to die at young ages due to pulmonary destruction. This is reflected in the relatively lower number of publications regarding hepatic and biliary involvement compared to pulmonary disease in CF. Furthermore, the limited number of cirrhotic pwCF (*n* = 4) examined in our cohort may not sufficiently represent the BA values in this subgroup. 

At the same time, our publication has the advantage of assessing a cohort naïve for CFTR-modulating therapies. Thus, it emphasizes the need to perform consecutive studies assessing the effects of CFTR modulators on bile homeostasis.

## 5. Materials and Methods

### 5.1. Participants and Settings

This prospective study was performed by recruiting pwCF of all ages (4–66 years) who were attended to between 2004–2005 at the CF Center of the Jena University Hospital, Germany. The study included *n* = 47 pwCF. The inclusion criteria were: (1) a diagnosis of CF determined by two positive sweat tests (sweat chloride of ≥30 mEq/L) and/or (2) detection of 2 disease-causing *CFTR* mutations with evidence of end organ involvement.

### 5.2. Ethical Statement

The study was approved by the Jena University ethics committee (registration number: 1222-11/03) and all methods were performed in accordance with the relevant guidelines and regulations. This study was conducted in strict accordance with the ethical guidelines in the Declaration of Helsinki. All pwCF and parents or guardians of minors provided written informed consent.

### 5.3. Measures of Clinical Data

BA analysis was performed with a modified method according to Sakakurah et al., 1998 [47]. A total of 10 free and 12 taurine- and glycine-conjugated bile acids were analyzed in the serum of pwCF using high performance liquid chromatography (HPLC) with post-column derivatization and fluorescence detection. 

A Jasco system (DG-1580-54, LG-1580-02, PU-1580, Ph-980, AS-1555, FP-1520-S, BORWIN Version1.50, Jasco, Tokyo, Japan) was used as chromatographic apparatus, and an Inertsil ODS-2 analytical column (5μm, 150 × 4.6 mm, GL Sciences Inc., Tokyo, Japan) was used for separation. A 3α-Hydroxysteroiddehydrogenase column (E-3α-HSD) from Sekisui Chemical Co., Ltd., Tokyo, Japan was used for post-column derivatization. Reagent β-Nicotinamide adenine dinucleotide (β-NAD) was obtained from Sigma (St. Louis, MO, USA). Methanol and acetonitrile were of HPLC-grade and all other reagents were of analytical grade. OASISTM3cc HLB cartridges were obtained from Waters Corporation Milford, MA, USA.

The following free and conjugated BAs were purchased from Steraloids (Wilton, NH, USA): cholic acid (CA), glycocholic acid (G-CA), taurocholic acid (T-CA), chenodeoxycholic acid (CDCA), glycochenodeoxycholic acid (G-CDCA), taurochenodeoxycholic acid (T-CDCA), deoxycholic acid (DCA), glycodeoxycholic acid (G-DCA), taurodeoxycholic acid (T-DCA), lithocholic acid (LCA), glycolithocholic acid (G-LCA), taurolithocholic acid (T-LCA), hyodeoxycholic acid (HDCA), glycohyodeoxycholic acid (G-HDCA), taurohyodeoxycholic acid (T-HDCA), hyocholic acid (HCA), glycohyocholic acid (G-HCA), taurohyocholic acid (T-HCA), α-murocholic acid (α-MCA), β-muricholic acid (β-MCA), murocholic acid (MOCA), and tauro-β-muricholic acid (T-β-MCA).

The established PIP score adapted from Ooi et al., as published in 2011 [25], was used to measure the severity of specific *CFTR* mutations in regard to pancreatic function. PwCF carrying mutations not included in the study by Ooi et al. [25] were excluded from the PIP–genotype analysis (8/47 pwCF); 4 pwCF’s *CFTR* mutations had not been identified and the mutations of 4 other pwCF had not been described in the PIP cohort from Ooi. et al. [25]. Mutations of pwCF with a PIP score ≤ 0.40 were classified as mild *CFTR* genotypes (*n* = 9), and those with a PIP score ≥ 0.88 as severe genotypes (*n* = 30). It is important to mention that these cutoffs differ from those originally described by Ooi et al. [25] (classified as either “mild” (≤0.25) or “severe” (>0.25) on the basis of the PIP score). As none of the included pwCF revealed PIP scores between 0.4 and 0.88, we excluded moderate as a classification and defined pwCF’s *CFTR* genotype severity as either mild or severe. Pancreatic insufficiency (PI) was defined as a clinical diagnosis by the need for pancreatic enzyme replacement therapy (PERT).

Ultrasound (US) examinations were performed in all pwCF and included the evaluation of 17 CF-relevant pathologies in abdominal US [36]. Furthermore, CFLD was determined retrospectively in 46 of the 47 pwCF, according to criteria defined in 2011 by Debray et al. [26]. Based on a consensus among hepatologists at a meeting of the North American CF Foundation in 2007, pwCF were classified into three categories: cystic fibrosis without evidence of liver disease (CF *w*/*o* LD) (*n* = 32), cystic fibrosis-related liver involvement without cirrhosis (CFLI *w*/*o* LC) (*n* = 10), and cystic fibrosis-related liver disease with cirrhosis (CFLD with LC) (*n* = 4) [4].

Biliary acid composition did not show any significant differences according to ursodeoxycholic acid (UDCA) administration. Therefore, UDCA and its conjugates were excluded from our analysis.

### 5.4. Data Analysis

All statistical analyses were performed using SPSS v.25.0 (IBM Corp. 2015, Version 25.0. Inc., Armonk, NY, USA). Normality in the distributions of the samples was tested using the Kolmogorov–Smirnov test. As all BA data samples failed to meet normality assumptions, Mann–Whitney U tests were performed to determine statistical differences between the medians of two independent samples. Results are reported as median and first and third quartiles (abbreviated as [Q_1_, Q_3_]) and are represented in boxplots. Pairwise correlations between variables were calculated using the Pearson’s correlation coefficient. A *p*-value ≤0.05 indicated a significant difference or correlation. Figures were created with GraphPad Prism version 8.4.3 for Windows, GraphPad Software, San Diego, CA, USA.

## 6. Conclusions

We assessed the concentrations of 22 BAs in the serum of pwCF, including primary, secondary, and tertiary BAs, as well as their respective glycine and taurine conjugates. Higher concentrations of total BAs were significantly associated with both *CFTR* genotype severity and pancreatic insufficiency. When measuring each BA individually, CA levels were significantly associated with more severe *CFTR* genotypes, as quantified by their PIP score for pancreatic insufficiency and non-cirrhotic CF-related liver involvement. Our study highlights the relevance of *CFTR* genotype severity in the assessment of enterohepatic circulation. Clinically, the improvement in BA homeostasis is a subject of high importance, as hepatobiliary involvement is the third most frequent reason of premature death in CF. In this regard, the assessment of BAs as potential surrogate markers when assessing the impact of highly effective CFTR modulator therapies on liver function may provide new insights into the pathophysiology of CFLD.

## Figures and Tables

**Table 1:** Demographic characteristics of the included people with CF (pwCF) (*n* = 47).

Variable		*n* (%)
Sex	Female	22 (46.8%)
	Male	25 (53.2%)
Genotype	F508del/F508del	18 (38.3%)
	F508del/other	20 (42.6%)
	G551D/other	4 (8.5%)
	unknown/unknown	3 (6.4%)
Age (years)	0–5	5 (10.6%)
	6–11	7 (14.9%)
	12–17	12 (25.5%)
	≥18	23 (48.9%)
Therapy	PwCF with PERT †	42 (89.4%)
	Ursodeoxycholic acid (UDCA)	28 (59.6%)
	PwCF without UDCA	14 (29.8%)
	PwCF with limited adherence to UDCA	5 (10.6%)
Comorbidities	Exocrine pancreatic insufficiency (EPI)	42 (89.4%)
	Cystic fibrosis liver disease (CFLD) *	14 (30.4%)
	CFLD without liver cirrhosis *	10 (21.7%)
	Liver cirrhosis *	4 (8.7%)
	Pancreatic lipomatosis **	38 (80.6%)
	PIP-Score ≥ 0.88 +	30 (76.9 %)
	PIP-Score ≤ 0.40 +	9 (23.1 %)

† Pancreatic enzyme replacement therapy (PERT). * Diagnostic criteria for CFLD were assessed in 46 of 47 pwCF. ** 45 of the 47 pwCF were screened for pancreatic lipomatosis. + Pancreatic insufficiency prevalence (PIP) scores were calculated for 39 of the 47 pwCF.

**Table 2:** Serum primary bile acids in pwCF classified according to their pancreatic insufficiency prevalence (PIP) score (median and [Q_1_, Q_3_]).

Serum Bile Acids (μmol/L) and Ratios	Mild *CFTR* Genotype (PIP ≤ 0.4)	Severe *CFTR* Genotype (PIP ≥ 0.88)	*p*
Free CA	0.12 (0.05, 0.28)	0.41 (0.19, 0.70)	0.009
G-CA	0.20 (0.09, 0.29)	0.29 (0.18, 0.71)	0.123
T-CA	0.03 (0.01, 0.06)	0.01 (0.01, 0.05)	0.315
Total CA	0.38 (0.27, 0.65)	1.01 (0.56, 1.37)	0.004
Free CDCA	0.08 (0.02, 0.29)	0.22 (0.08, 0.49)	0.069
G-CDCA	0.26 (0.15, 0.52)	0.65 (0.29, 1.06)	0.086
T-CDCA	0.14 (0.05, 0.18)	0.07 (0.03, 0.14)	0.169
Total CDCA	0.54 (0.27, 0.97)	1.12 (0.47, 1.59)	0.123
Total free primary BAs	0.15 (0.07, 0.54)	0.65 (0.32, 1.30)	0.020
Total primary BAs	0.84 (0.59, 1.57)	2.04 (1.24, 2.74)	0.025
Glycine-conjugated BAs	0.51 (0.20, 0.74)	1.03 (0.48, 1.63)	0.069
Taurine-conjugated BAs	0.14 (0.09, 0.25)	0.09 (0.04, 0.19)	0.180
Free CA / Free CDCA	1.05 (0.65, 3.15)	2.01 (0.93, 3.80)	0.299
Total CA / Total CDCA	0.66 (0.36, 0.95)	0.79 (0.58, 1.59)	0.131
T-CA / T-CDCA	0.38 (0.15, 0.62)	0.30 (0.11, 0.64)	0.755
Conj. CA / Conj. CDCA	0.48 (0.29, 0.93)	0.60 (0.36, 0.85)	0.566
G:T ratio (including all BAs)	2.40 (1.70, 6.60)	7.40 (5.50, 9.90)	0.008
Total BAs	1.25 (0.93, 2.19)	2.62 (1.68, 3.59)	0.033

CA: cholic acid; G-CA: glycocholic acid; T-CA: taurocholic acid; CDCA: chenodeoxycholic acid; G-CDCA: glycochenodeoxycholic acid; T-CDCA: taurochenodeoxycholic acid; BA: bile acid; Conj.: conjugated; G: glycine; T: taurine.

**Table 3:** Secondary bile acids distribution in different subgroups classified according to their *CFTR* genotype (median and [Q_1_, Q_3_]).

Serum Bile Acids (nmol/L)	PIP ≤ 0.4	PIP ≥ 0.88	*p*
DCA	0.17 (0.04, 0.32)	0.13 (0.03, 0.23)	0.731
G-DCA	0.11 (0.05, 0.14)	0.06 (0.02, 0.14)	0.402
T-DCA	0.01 (0.01, 0.02)	0.01 (0.0, 0.02)	0.454
LCA	0.0 (0.0, 0.01)	0.01 (0.0, 0.01)	0.238
G-LCA	0.01 (0.0, 0.03)	0.01 (0.01, 0.02)	0.805
T-LCA	0.01 (0.0, 0.02)	0.02 (0.0, 0.05)	0.183

DCA: deoxycholic acid; G-DCA: glycodeoxycholic acid; T-CA: taurodeosycholic acid; LCA: lithocholic acid; G-LCA: glycolithocholic acid; T-LCA: taurolithocholic acid.

**Table 4:** Median concentrations of serum biochemical parameters alanine aminotransferase (ALT), aspartate aminotransferase (AST), γ-glutamyl transpeptidase (γ-GT), alkaline phosphatase (AP), and glutamatdehydrogenase (GLDH) (median and [Q_1_, Q_3_]).

	Mild Genotype Median [Q_1_, Q_2_]	Severe Genotype Median [Q_1_, Q_2_]	*p*	Without CFLD Median [Q_1_, Q_2_]	With CFLD Median [Q_1_, Q_2_]	*p*
ALT (µmol/L)	0.37 (0.28, 0.44)	0.50 (0.28, 0.76)	0.15	0.38(0.25, 0.47)	0.71 (0.37, 0.78)	0.005
AST (µmol/L)	0.30 (0.21, 0.42)	0.42 (0.32, 0.58)	0.02	0.35 (0.24, 0.47)	0.48 (0.34, 0.66)	0.033
γ-GT (µmol/L)	0.22 (0.17, 0.36)	0.25 (0.13, 0.49)	0.90	0.17 (0.13, 0.26)	0.50 (0.34, 0.74)	0.0002
AP (µmol/L)	1.64 (1.27, 2.03)	2.53 (1.54, 4.03)	0.02	1.77 (1.31, 3.18)	3.68 (1.69, 4.14)	0.039
GLDH (µmol/L)	44.0 (20.0, 50.0)	50.0 (28.5, 114.5)	0.33	44.0 (25.5, 52.0)	62.5 (44.0, 115.0)	0.036

**Table 5:** Serum bile acids distribution in different subgroups classified according to their pancreatic sufficiency status (median and [Q_1_, Q_3_]).

Serum Bile Acids (μmol/L) and Ratios	PS (*n* = 5)	PI (*n* = 42)	*p*
Free CA	0.13 (0.05, 0.35)	0.27 (0.15, 0.57)	0.110
G-CA	0.12 (0.03, 0.25)	0.25 (0.15, 0.71)	0.064
T-CA	0.01 (0.00, 0.04)	0.02 (0.01, 0.05)	0.495
Total CA	0.35 (0.17, 0.50)	0.83 (0.40, 1.28)	0.034
Free CDCA	0.15 (0.01, 0.24)	0.23 (0.08, 0.44)	0.146
G-CDCA	0.18 (0.15, 0.31)	0.56 (0.24, 1.06)	0.025
T-CDCA	0.14 (0.09, 0.25)	0.08 (0.03, 0.16)	0.190
Total CDCA	0.44 (0.26, 0.79)	0.97 (0.47, 1.59)	0.045
Total free primary BAs	0.38 (0.07, 0.53)	0.56 (0.31, 0.88)	0.095
Total primary BAs	0.97 (0.43, 1.21)	1.91 (1.01, 2.74)	0.023
Glycine-conjugated BAs	0.39 (0.17, 0.51)	0.90 (0.43, 1.63)	0.034
Taurine-conjugated BAs	0.14 (0.10, 0.29)	0.10 (0.04, 0.22)	0.202
Free CA/Free CDCA	3.16 (0.74, 3.94)	1.35 (0.80, 3.17)	0.651
Total CA/Total CDCA	0.71 (0.27, 1.20)	0.71 (0.54, 1.25)	0.535
T-CA/T-CDCA	0.04 (0.02, 0.43)	0.33 (0.13, 0.64)	0.215
Conj. CA/Conj. CDCA	0.19 (0.09, 0.93)	0.51 (0.36, 0.81)	0.354
G:T (including all BAs)	2.40 (1.40, 3.20)	6.60 (4.20, 9.30)	0.002
Total BAs	1.38 (0.62, 1.80)	2.53 (1.34, 3.59)	0.034

CA: cholic acid; G-CA: glycocholic acid; T-CA: taurocholic acid; CDCA: chenodeoxycholic acid; G-CDCA: glycochenodeoxycholic acid; T-CDCA: taurochenodeoxycholic acid; PS: pancreatic sufficiency; PI: pancreatic insufficiency.

**Table 6:** Serum bile acids distribution in different subgroups classified according to CF-related liver involvement (median and [Q_1_, Q_3_]).

Serum Bile Acids (μmol/L) and Ratios	CF without LI (*n* = 32)	CFLI without LC (*n* = 10)	CFLI with LC (*n* = 4)	p1	p2	p3
Free CA	0.25 (0.11, 0.46)	0.56 (0.26, 0.50)	0.30 (0.11, 0.74)	0.036	0.827	0.454
G-CA	0.21 (0.14, 0.57)	0.25 (0.06, 0.84)	0.52 (0.20, 0.70)	0.988	0.480	0.733
T-CA	0.01 (0.01, 0.06)	0.01 (0.00, 0.05)	0.03 (0.02, 0.05)	0.722	0.315	0.304
Total CA	0.66 (0.32, 1.09)	1.04 (0.52, 1.44)	1.01 (0.37, 1.29)	0.108	0.680	0.635
Free CDCA	0.24 (0.08, 0.41)	0.16 (0.07, 0.46)	0.26 (0.05, 0.47)	0.896	0.903	1.000
G-CDCA	0.40 (0.26, 1.01)	0.45 (0.15, 1.64)	0.67 (0.28, 0.84)	0.965	0.903	0.839
T-CDCA	0.10 (0.05, 0.17)	0.06 (0.03, 0.17)	0.05 (0.02, 0.13)	0.328	0.173	0.733
Total CDCA	0.82 (0.49, 1.54)	0.84 (0.31, 2.22)	0.98 (0.36, 1.43)	0.988	0.827	0.635
Total free primary BAs	0.54 (0.26, 0.79)	0.68 (0.33, 1.45)	0.59 (0.17, 1.18)	0.192	0.865	0.539
Total primary BAs	1.64 (1.04, 2.51)	2.30 (0.82, 3.98)	2.05 (0.73, 2.65)	0.551	0.981	0.539
Glycine-conjugated BAs	0.71 (0.48, 1.41)	0.70 (0.20, 2.68)	1.19 (0.48, 1.54)	0.965	0.645	0.839
Taurine-conjugated BAs	0.11 (0.08, 0.24)	0.10 (0.03, 0.19)	0.08 (0.04, 0.18)	0.512	0.269	0.839
Free CA / Free CDCA	1.12 (0.69, 3.21)	3.05 (0.95, 3.64)	2.01 (1.11, 2.16)	0.172	0.645	0.188
Total CA / Total CDCA	0.61 (0.49, 1.09)	1.20 (0.57, 1.86)	1.03 (0.83, 1.16)	0.115	0.115	0.635
T-CA / T-CDCA	0.29 (0.06, 0.50)	0.25 (0.08, 0.39)	0.69 (0.43, 2.43)	0.657	0.038	0.036
Conj. CA / Conj. CDCA	0.50 (0.32, 0.84)	0.43 (0.28, 0.83)	0.78 (0.70, 0.84)	0.988	0.157	0.240
G:T (including total BAs)	6.20 (2.30, 8.50)	6.70 (3.60, 15.30)	5.80 (5.10, 11.60)	0.130	0.543	0.839
Total BAs	2.06 (1.37, 3.22)	2.67 (1.13, 4.97)	2.70 (1.08, 3.65)	0.611	0.134	0.240

CF: cystic fibrosis; LI: liver involvement; CFLI: cystic fibrosis with liver involvement; LC: liver cirrhosis; CA: cholic acid; G-CA: glycocholic acid; T-CA: taurocholic acid; CDCA: chenodeoxycholic acid; G-CDCA: glycochenodeoxycholic acid; T-CDCA: taurochenodeoxycholic acid. p1: CF without LI vs. CFLI without LC groups. p2: CF without LI vs. CFLI with LC groups. p3: CFLI without LC vs. CFLI with LC groups.

## Data Availability

The datasets generated during and/or analyzed during the current study are available from the corresponding author upon reasonable request.
